# The Reorganised American Medical Association

**Published:** 1902-07

**Authors:** 


A Monthly Review of Medicine and Surgery.

*EDITOR:*

WILLIAM WARREN POTTER, M. D.

All communications, whether of a literary or business nature, books for review and exchanges should be addressed to the editor: 284 Franklin Street, Buffalo, N. Y.

Vol. XLI.—LVII. JULY, 1902. No. 12.

### The Reorganised American Medical Association.

THE first meeting of the American Medical Association with a new constitution and by-laws was held at Saratoga Springs, June 10-13, 1902, under the presidency of Dr. John Allan Wyeth, of New York. The convention was well attended though the number registered was smaller than for several years past. The cool weather that prevailed during the week contributed to make the section work of a higher order than usual.

The chief interest grouped around the house of delegates because of the novelty of that body and the curiosity pertaining to its first legislative work. If there were those who predicted its failure or hoped for its confusion, they were doomed to disappointment; for it must be confessed that looked at from all sides and from every viewpoint, and considered with reference to its lack of experience and the somewhat heterogeneousness of its makeup, its first legislative season closed with harmony, after the performance of a large amount of valuable work that will serve to advance the welfare of the association. If, again, there were those who were determined that the plan should be abandoned after this one trial, and a return made to the old methods of procedure, they, too, are destined to disappointment because the system is likely to endure. That there will be occasion for changes in detail is probable, but the general structure is likely to last for many years.

Another feature of interest centered in the relationship of the Medical Society of the State of New York to the Association. The former is the parent organisation, though it was excommunicated from the latter twenty years ago, by reason of its heresy regarding the code of ethics. It had modified this very ancient and honorable document in conformity with the requirements of law. It boldly took a step in the open that others were taking in secret. It obeyed the majesty of the law and was punished by the exponents of the gospel,—for the chief priests and elders persuaded the multitude. Happily, however, the heresy of 1882 has become the orthodoxy of 1902, as is so truthfully and cleverly stated by *The Medical Record;* hence the unification of the medical profession is believed to be near at hand.

Acting upon the suggestion which grew out of a conference of the leaders of the opposing factions, the house of delegates authorised the appointment of a committee to consider and report a protocol as a basis of union, which is to be presented next year. President Wyeth appointed as such committee: E. Elliott Harris and Joseph D. Bryant, of New York; William H. Welch, of Baltimore; Nicholas Senn, of Chicago, and T. J. Happel, of Trenton, Tennessee. Every reference made to unification in any of the addresses or remarks from the platform was greeted with hearty cheers, indicating that the rank and file are already anxious for the final act to be promulgated, that shall restore harmony and good fellowship into the ranks of a learned profession where naught else should prevail.

Taking up the spirit of progress that is everywhere manifested by higher resolves and loftier purposes, President Wyeth concluded his address with an eloquent appeal to the better judgment and purer motives of the members, from which we quote as follows:

The American Medical Association is the sponsor for organised medicine in the United States, and failure to accomplish this end implies the failure of this Association. We must not fail, nor will we, unless we falter in carrying out the plan of reorganisation in the liberal and progressive spirit which characterised the session of 1901. It is our plain duty to endeavor to bring about the adoption by the various constituent bodies of a practically uniform constitution and by-laws for each county, and for each state, modified only as the local conditions may require, and all governed by the same rules of conduct. These rules, as at present given in the code of ethics, adopted many years ago by this association, should be also a subject of serious consideration at this time, for we can not claim consistency or be logical in argument until there is but one code for the national association and for all of the state organisations represented in the national body. This, as you well know, does not prevail. Some years ago there lived and labored among us for the good of mankind and the honor of the profession a man whose genius was of the highest order, and whose name carried the fame of American surgery throughout the world. He was one of those fearless pioneers in science who found his place ever on the frontier clearing the way for those who were to follow. In 1876, at the meeting of the American Medical Association in Philadelphia, Dr. J. Marion Sims, in his presidential address, referring to the code of ethics, says: "The time will come when your organic laws, like the constitution of our country, will require modifications and amendments to suit a higher intelligence, a broader education and a greater destiny." In my opinion, the time has come when we can not absolve ourselves from the responsibility of doing away with the inconsistencies for which we may now be properly criticised.

Such have been the changes in the statutes of a majority of the states since the code was adopted by the respected founders of this association, that we find it insisting upon conduct on the part of our members which is contrary to the laws of the states in which they reside. For instance, one section forbids a member of the regular profession to act upon a board of examiners which has to pass upon the legal qualifications of persons not graduates of regular medical colleges, while in thirty-eight of the states represented here, the civil statutes require these boards, which are composed in great part of members of the association, to examine, pass upon and sign certificates or licenses to practise of homeopaths, eclectics, and other subdivisions of medical practice. In six of these states, including the District of Columbia, the law requires three separate examining boards. In Mississippi, North Carolina and South Carolina the examining boards are entirely composed of regular physicians, and in one of these (Mississippi), while none but regulars are allowed on the board, the law explicitly says: "Distinction shall not be made between applicants because of the different systems or schools of practice that may be chosen." In almost all the states and territories regular physicians are compelled by the laws of the state in which they reside to disobey the injunctions of this section of the code of ethics.

A modification of this and other sections of the code must be a part of the liberal plan of reorganisation which we have essayed.

In conclusion, I ask this association to stand for more than the healing art. To labor for the alleviation of suffering and for the restoration of health is a noble avocation, but to teach our fellows how to avoid disaster is a prouder privilege and a higher duty. We should be teachers of men. How better can we protect the public from disease in all its various forms and insidious processes than by perfecting in every county and in every community an organisation which shall be ever watchful and insistent upon obedience to the laws relating to the public health?

These lofty thoughts clothed in singularly fitting terms were received with an outburst of applause that spoke in an unmistakable manner of their welcome by the large audience that heard them, and as they are read by the countless thousands throughout the length and breadth of the land, they will further receive the gratifying indorsement "well done good and faithful servant!"

May we not expect that the good work begun by Charles A. L. Reed, and carried on by John Allan Wyeth, will be brought to a successful conclusion by Frank Billings?

